# A Selective Iodide Ion Sensor Electrode Based on Functionalized ZnO Nanotubes

**DOI:** 10.3390/s130201984

**Published:** 2013-02-04

**Authors:** Zafar Hussain Ibupoto, Kimleang Khun, Magnus Willander

**Affiliations:** Department of Science and Technology, Campus Norrköping, Linköping University, SE-60174 Norrköping, Sweden; E-Mails: kimleang.khun@liu.se (K.K.); magnus.willander@liu.se (M.W.)

**Keywords:** ion selective electrode, ZnO nanotubes, miconazole nitrate, potentiometric technique, potentiometric titration

## Abstract

In this research work, ZnO nanotubes were fabricated on a gold coated glass substrate through chemical etching by the aqueous chemical growth method. For the first time a nanostructure-based iodide ion selective electrode was developed. The ZnO nanotubes were functionalized with miconazole ion exchanger and the electromotive force (EMF) was measured by the potentiometric method. The iodide ion sensor exhibited a linear response over a wide range of concentrations (1 × 10^−6^ to 1 × 10^−1^ M) and excellent sensitivity of −62 ± 1 mV/decade. The detection limit of the proposed sensor was found to be 5 × 10^−7^ M. The effects of pH, temperature, additive, plasticizer and stabilizer on the potential response of iodide ion selective electrode were also studied. The proposed iodide ion sensor demonstrated a fast response time of less than 5 s and high selectivity against common organic and the inorganic anions. All the obtained results revealed that the iodide ion sensor based on functionalized ZnO nanotubes may be used for the detection of iodide ion in environmental water samples, pharmaceutical products and other real samples.

## 1. Introduction

Iodine is one of the important constituents among other human nutrients due to it's key contributions in many biological pathways such as enrichment of brain development, metabolism, neurological functions and thyroid gland activity, *etc.* The cell growth of humans and animals is dependent on the supply of iodine. In those places where water and soil have substandard levels of iodine, people are suggested to take extra iodine in the diet to maintain a normal level of iodine. Due to low levels of iodine, diseases like iodine deficiency disorder (IDD) can occur [1], whereas high levels of iodine in the diet can lead to many pathological ailments. Many iodine compounds are widely used in the synthesis of pharmaceutical products in order to treat the deficiency of iodine. The iodine compounds are also used as antiseptics and disinfectants. Iodine is found in trace quantities in samples such as foodstuffs [2,3], fodder [4], clinical and biological species [4,5], synthetic pharmaceuticals [6,7], the environment [8,9] and industrial samples [10]. After a survey of the literature, it was found that numerous analytical techniques have been employed for the detection of trace quantities of iodine in analysed samples. Those techniques include ion chromatography [10], chemiluminescence [5], diffuse reflectance spectroscopy [11], inductively coupled plasma-atomic emission spectroscopy [12], flow-injection analysis [13], *etc.* These analytical tools have some limitations such as consuming high volume of analyte, and taking a long time for analysis, but this is not true for all the techniques such as carrier-based ion-selective electrodes that have been used to a large extent for normal analysis. The development of ion selective electrodes for the iodide ion based on the ionophore without ion exchangers or precipitation have been reported [14]. Over the last 20 years, a number of papers on iodide ion sensors have been published based on the different derivatives such as Mn (IV) [15,16], Co (III) [17,18], Sn (IV) [19,20], Mo (V) [21], porphyrin complexes, Co (II), Cu (II) phthalocyanine derivatives [22], Hg (II) complex [23,24], and Co (II) triazole derivative [25], *etc.* Many of these iodide ion selective electrodes have some working limitations such as low linearity, slow response times and high limits of detection for the target electrolyte. These iodide ion selective electrodes also interfere with lipophilic anions and few of them have shown excellent working activity. The potentiometric technique, on the other hand, has many advantages like simple analysis, fast response, low cost, high sensitivity and selectivity towards the analyte.

The application of nanomaterials in sensing has opened a new gateway for the engineering of transducers in sensor science with high sensitivity, selectivity and fast response times. The biosensors based on the nanomaterials have excellent performance due to their sub-micrometres and nano- dimensions. A highly sensitive platinum-based glucose biosensor had been reported [26]. Among other nanostructures of ZnO, one dimensional (1D) nanostructures have outstanding applications in optics, optoelectronics, sensors, and actuators because of their excellent semiconducting and piezoelectric characteristics [27–30]. There are many pathways for the use of ZnO nanostructures in the area of biosensing because of the significant options for functionalization with enzymes/ionophores. Because of the high surface to volume ratio of one dimensional ZnO nanostructures, nanosensors based on these nanostructures show strong electrical signals, better catalytic response and fast flow of electrolyte via the sensor. A number of efforts has been undertaken to fabricate different types of chemical and biochemical sensors composed of ZnO nanomaterials like nanoscale ZnO-based fluorescent biosensors [31,32], sensors for hydrogen sulphide (H_2_S) gas based on single ZnO nanowires [33], intracellular pH sensors based on ZnO nanorods [34], and ethanol sensors based on flowerlike ZnO nanostructures [35]. ZnO nanotubes possess many attractive properties like high porosity and surface to volume ratio as compared to those of ZnO nanorods, nanowires, nanobelts, *etc.* and show better performance than other one dimensional nanostructures [36–38]. Moreover, ZnO nanostructures are also nontoxic, bio- safe, biocompatible, fast in electron communication, high sensitive to target analytes, cheap and also easily fabricated. In this work, we have fabricated the ZnO nanotubes on the gold coated glass substrate and functionalized these nanotubes with ion exchanger miconazole nitrate. The iodide ion sensor has shown low limit of detection, fast response time, high sensitivity, selectivity and stability.

## 2. Experimental

### 2.1. Reagents

The miconazole nitrate, dioctylsebacate (DOS), *o*-nitrophenyl octyl ether (NPOE), dioctylphathalate (DOP), cetyltrimethyl ammonium bromide (CTAB), tetrahydrofuran (THF), high molecular weight polyvinyl chloride (PVC), silver nitrate (AgNO_3_), salts of potassium and sodium were purchased from Sigma Aldrich (Stockholm, Sweden). The analyte solution was prepared in deionized water. Other chemicals used were of analytical grade.

### 2.2. Fabrication and Growth of ZnO Nanotube Arrays

**Figure 1.**
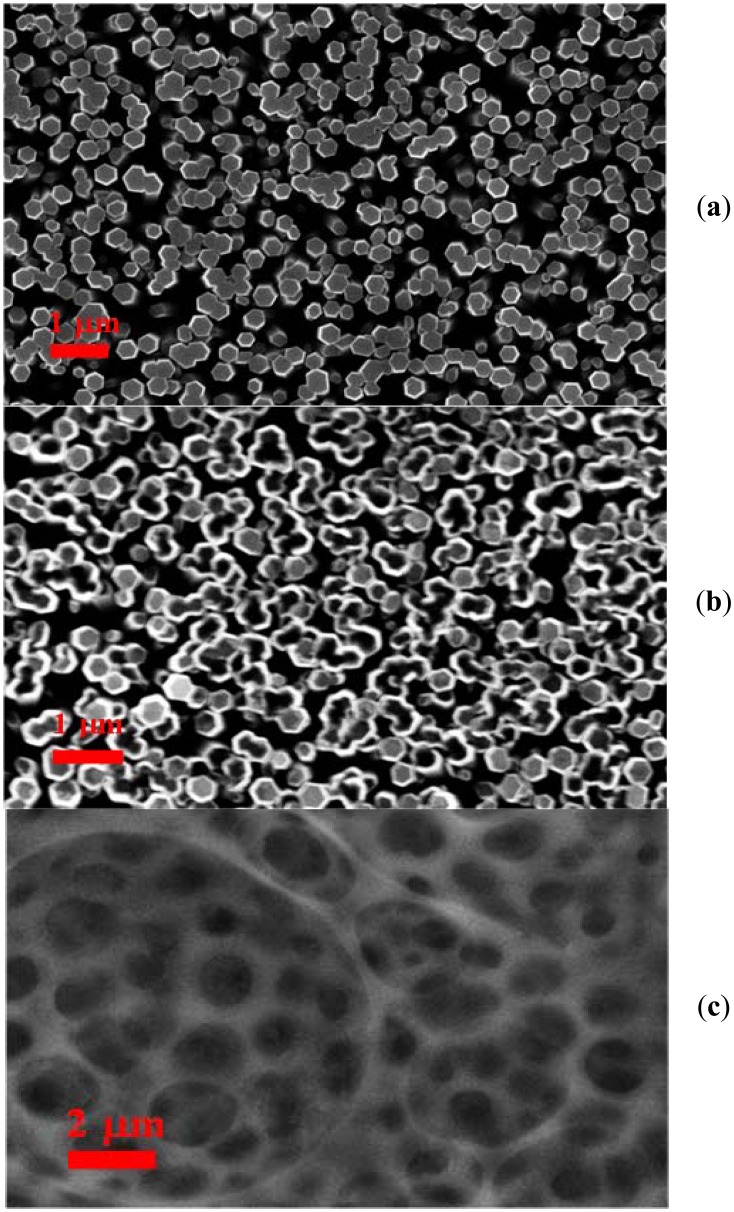
(**a**) FESEM image of ZnO nanorods grown on a gold coated glass substrate, (**b**) FESEM image of ZnO nanotubes after chemical etching, and (**c**) FESEM image of the functionalized ZnO nanotubes.

The process of fabrication of ZnO nanotubes on the gold coated glass substrates was as follows: The borosilicate glass substrates were affixed in the vacuum chamber of a Satis (CR 725) evaporator. Afterwards an adhesive layer of 30 nm thickness of titanium was deposited, and then 100 nm thickness layer of gold was evaporated. The fabrication of ZnO nanotubes was performed by a two steps chemical method. In the first step ZnO nanorods were prepared by the low temperature hydrothermal growth method [37]. The morphology of the as-grown ZnO nanorods was studied using the field emission scanning electron microscopy (FESEM) technique. It was observed that the prepared ZnO nanorods were highly aligned and dense, with a diameter of 100 nm to 200 nm, as shown in Figure 1(a). In the next step, the ZnO nanorods were chemically etched into ZnO nanotubes using a described method [39,40]. The grown ZnO nanorods were fixed in a Teflon sample holder and kept in 3.5 to 5.5 M solution of potassium chloride (KCl). The conversion of ZnO nanorods into nanotubes is dependent on different reaction parameters like the concentration of potassium chloride, temperature, and time. The experiment was repeated several times in order to obtain the desired ZnO nanotubes with high etching throughout the substrates, as shown in Figure 1(b). It can be inferred from the Figure 1(b) that ZnO nanotubes are well etched and exhibit good nanotube-like morphology.

### 2.3. Functionalization of ZnO Nanotubes with Ion Exchanger Miconazole Nitrate

It has been already reported that the miconazole ion exchanger has great affinity for the iodide ion, producing an addition product [41]. In the reported work Jalali *et al.* [41] used the same ion exchanger with a PVC membrane and the composition of the membrane was described as: 33 mg of PVC, 2 mg of miconazole nitrate dissolved in a small quantity of methanol, 2 mg of CTAB, and 63 mg of DOS as plasticizer and finally the mixture was dissolved in 3 mL of THF. A Teflon tube of (3–5 mm o.d) was used for the formation of a 0.3 mm film of membrane mixture on the tube. In the present work, taking advantage of the ZnO nanomaterial, miconazole was also selected for the development of a potentiometric ion selective electrode based on ZnO nanotubes.

A solution of miconazole nitrate (2 mg) was prepared in a few drops of methanol, and then mixed with PVC (160 mg), DOS (63 mg), CTAB (2 mg), and dissolved in THF (3 mL) as solvent. This composition was obtained after optimizing the different amounts of each constituent of the composition because the various components of the membrane solution have a significant effect on the performance and stability of the ion selective electrode, and especially PVC has shown a considerable effect in the present case. After the preparation of the optimized mixture of the abovementioned constituents, the ZnO nanotube-based electrodes were manually dipped into this mixture for a few minutes. The dipped ZnO nanotube-based electrodes were dried at room temperature for 1 hour, then kept for overnight at 4 °C. A FESEM image of the functionalized ZnO nanotubes is shown in Figure 1(c), which shows the filling of nanotubes with membrane solution and also that the surface of the ZnO nanotubes is covered with membrane. The membrane solution can be used for many days, if it can be kept under suitable conditions in order to avoid reactions with the atmosphere. The electrochemical response of the functionalized ZnO nanotube-based ion selective electrode was measured by the potentiometric method. In the constructed electrochemical cell assembly the functionalized ZnO nanotubes were used as working electrode and silver-silver chloride (Ag/AgCl) was used as reference electrode. The ZnO nanotube-based iodide ion selective electrodes were kept at 4 °C, when not in use.

### 2.4. Potentiometric Measurement

The potentiometric measurement and time response was measured using a Metrohm pH meter model 728 and a Keithley 2400 an electrical instrument, respectively. It is known that the ion selective electrodes respond to the ionic activity, and in doing so the potential is solely related to the concentration of an electrolyte. It is also very important that as the activity coefficient depends on these factors, they should be kept constant. Therefore during the experiments, in all solutions of iodide used 1 × 10^−3^ M potassium nitrate (KNO_3_) was added for the adjustment of the ionic strength of the test solution, but in the interference and pH experiments the potassium nitrate was not adjusted. All the experiments were performed at room temperature (23 °C), except those performed to study the effect of temperature on the response of ion selective electrode.

## 3. Results and Discussion

**Figure 2.**
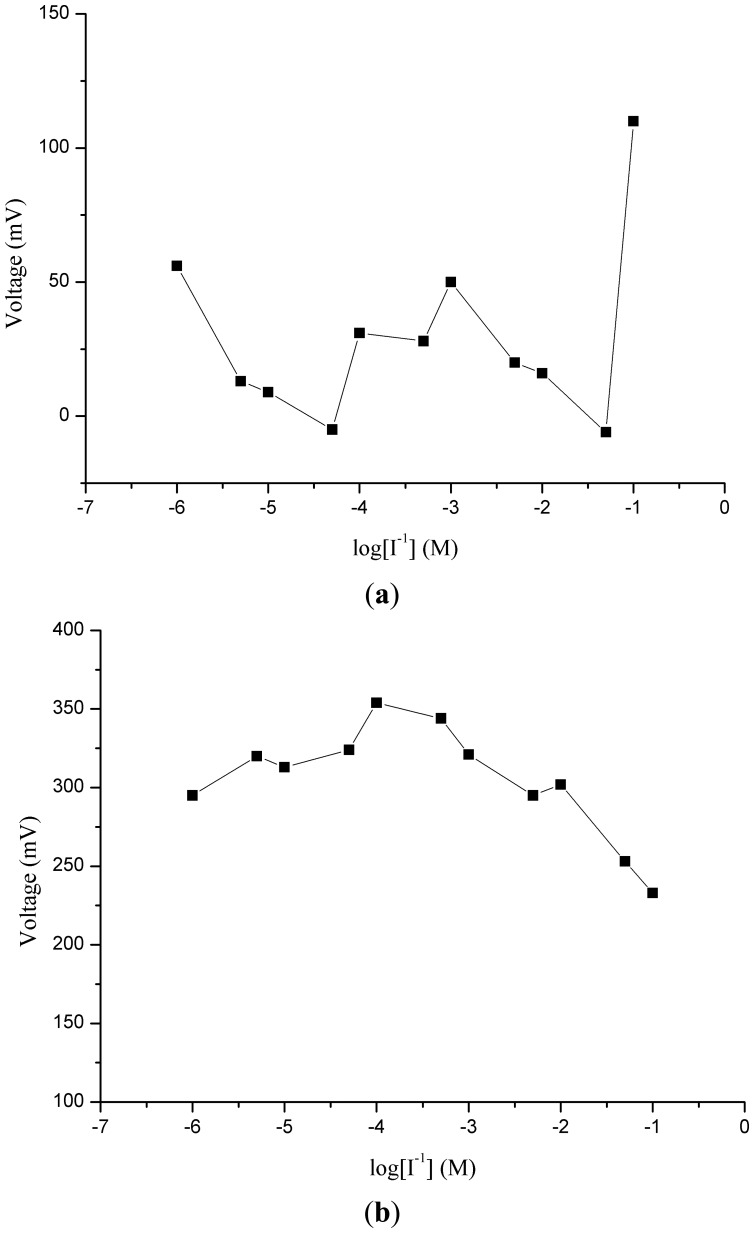

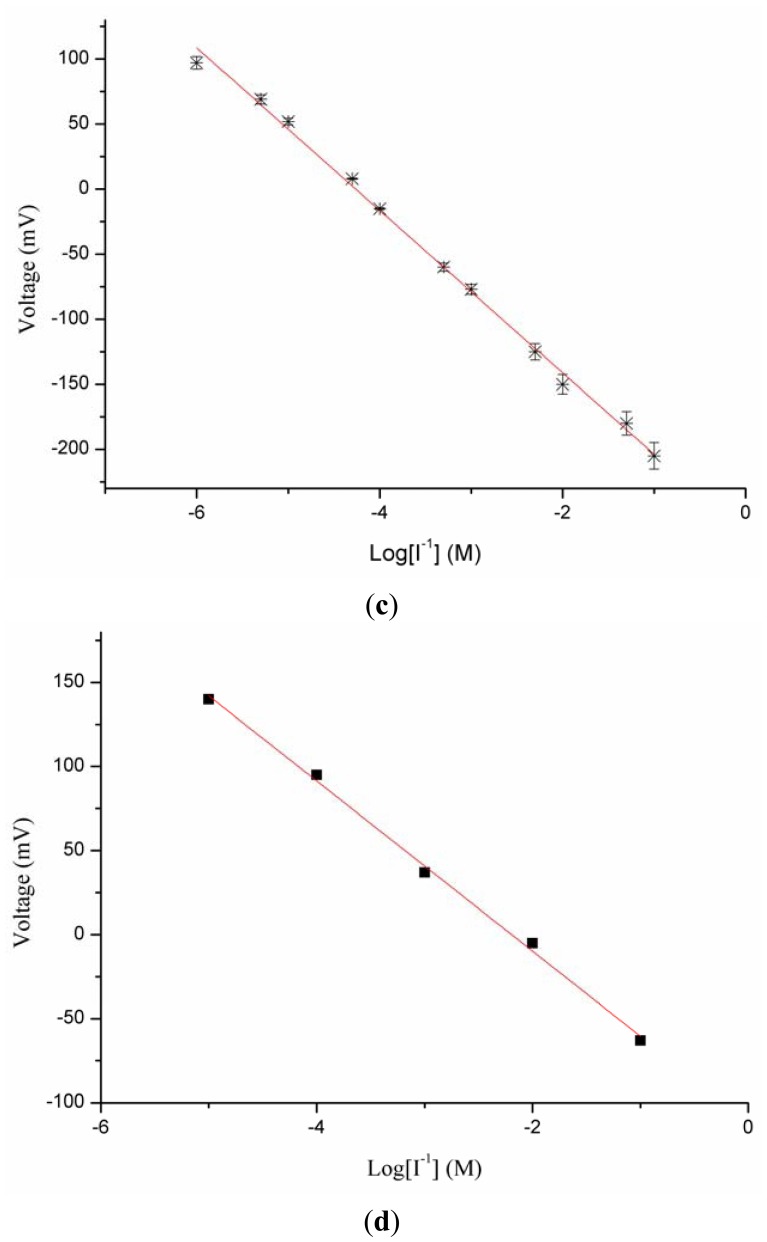
(**a**) The calibration curve of membrane paste of ion exchanger on the gold coated glass for 1 × 10^−6^−1 × 10^−1^ M concentration of iodide ion, (**b**) the calibration curve of ion exchanger paste on the gold coated silicon for 1 × 10^−6^−1 × 10^−1^ M concentration of iodide ions, (**c**) the calibration curve of selective iodide ion sensor based on the functionalized ZnO nanotubes for 1 × 10^−6^−1 × 10^−1^ M concentration of iodide ion, and (**d**) the calibration curve of selective iodide ion sensor based on the functionalized ZnO nanorods for 1 × 10^−5^−1 × 10^−1^ M concentration of iodide ions.

Potentiometric ion sensors with high selectivity and sensitivity, wide dynamic range of detection for the target analyte and fast response times are in high demand for routine analysis. In order to understand the role of ZnO nanotubes in the sensing mechanism, the same composition was pasted on gold coated glass and silicon electrodes. It can be seen from Figures 2(a,b) that the response of miconazole nitrate was found to be random and nonlinear. The reason for this behavior could be the loose attachment of the ion exchanger with the flat gold surface. This can also be inferred from Figure 2(c), which shows the potentiometric response of the iodide ion selective electrode based on the functionalized ZnO nanotubes with the ion exchanger miconazole nitrate. The iodide ion selective electrode based on the ZnO nanotubes demonstrated a highly linear range of detection from 1 × 10^−6^ M to 1 × 10^−1^ M for iodide anion with a good sensitivity of −62 ± 1 mV/decade and a regression coefficient of 0.99. The wide dynamic range of detection of iodide anion using the ZnO nanotube-based ion sensor confirmed the excellent performance of ZnO nanostructures due to the firm binding of miconazole membrane mixture on the surface of ZnO nanotubes. ZnO nanotubes exhibit hollow nanostructures which can carry a large number of miconazole ion exchanger membrane molecules and consequently a wide range of iodide ions is detected by the proposed selective iodide ion sensor. In order to understand the role of ZnO nanotubes, a comparative study was also carried by using the functionalized ZnO nanorods as shown in Figure 2(d). The functionalized ZnO nanorod-based iodide ion sensor showed a linear range from 1×10^−5^ to 1×10^−1^ M for iodide ion concentrations with a sensitivity of 51 ± 1 mV/decade. From this comparison it could be concluded that ZnO nanotubes having hollow structures compared to nanorods provide more surface area for the attachment of miconazole ion exchanger molecules which allows detection of more iodide ions, thus showing higher sensitivity and a lower limit of detection.

**Figure 3.**
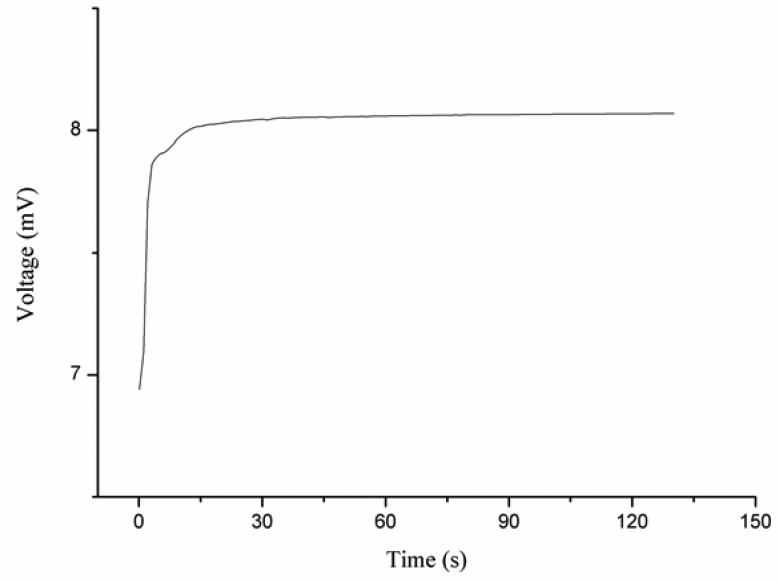
The response time of the iodide ion sensor for 1 × 10^−6^ M potassium iodide solution.

When the iodide ion sensor was tested in solution of iodide ion below 1 × 10^−6^ M, the response of the sensor was observed to be stable, but it was out of the linear range, so the detection limit was found to be 5 × 10^−7^ M. The effect of the volume of iodide ion solution on the output response of the ion sensor was also examined. The sensor showed no significant change in the output voltage for different volumes of the iodide solutions. The time response of the proposed iodide ion sensor was measured in different concentrations of iodide ion, and it was noticed that the sensor responded after about 10 s for 1 × 10^−6^ M iodide concetration. When the iodide ion sensor based on the functionalized ZnO nanotubes was employed in higher concentrations of iodide ion, the response time was found to be slightly less than 5 s, as shown in Figure 3.

**Figure 4.**
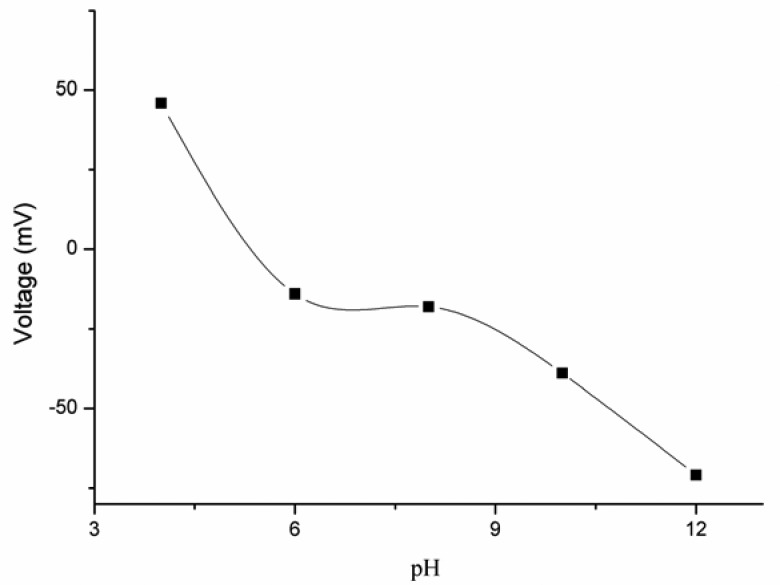
The effect of pH on the EMF response of the iodide ion sensor in 5 × 10^−3^ M potassium iodide solution.

The effect of different pH values of 5 × 10^−3^ M potassium iodide solution on the potential response of the iodide ion sensor electrode was studied for pH values ranging from 4 to 12. The pH of the iodide ion solutions was adjusted by adding 1 × 10^−1^ M sodium hydroxide (NaOH) and 1 × 10^−1^ M hydrochloric acid (HCl). It was observed that at lower pH values the EMF was higher, but from pH 6 to 8 the EMF response was found constant, and at higher pH values the iodide ion sensor showed a dramatic change in the output response due to simultaneous sensing of iodide (I^−^) and hydroxide (OH^−^) by the ZnO nanotube-based iodide ion sensor, as shown in Figure 4. This behavior of simultaneous detection of iodide and hydroxide ion was also already described in the published work on the miconazole nitrate ion exchanger [41].

**Figure 5.**
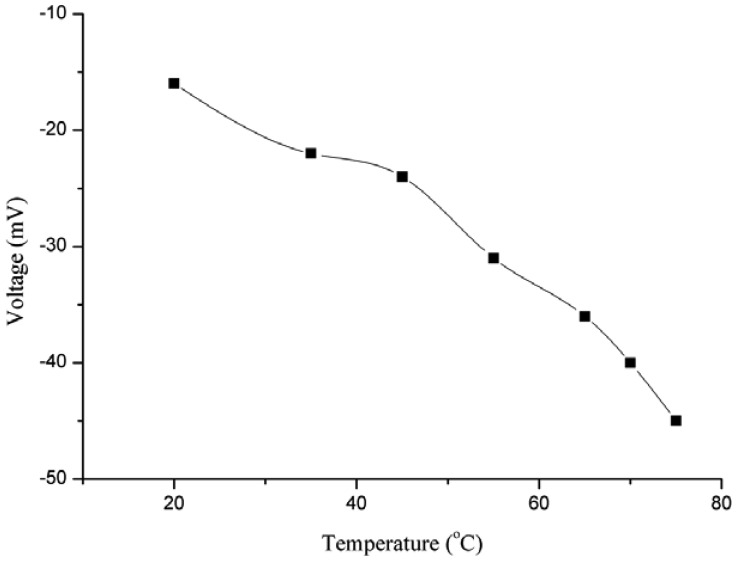
The temperature effect on the potential response of the iodide ion sensor.

Temperature also has an influence on the EMF response of ion selective electrodes. Because of the different temperatures, the ionic mobility of the ions which are to be analysed is different, so the potential should change. For the study of the effect of temperature on the output response, the temperature was changed in steps of 10 °C. The range of temperature effect to be studied was selected from room temperature (23 °C) to 75 °C, as shown in Figure 5. The potential response was observed to increase up to 45 °C, but afterwards the response decreased because at the first the increase in output response was due to the increase in the ionic mobility of iodide ion and later at higher temperatures the decreased response might be due to two factors: the first is the high ionic resistance offered at high temperature and second may be the detachment of the miconazole ion from the surface of the ZnO nanotubes.

The response of an anion selective electrode for the primary anion over other anions in test solutions is very crucial. This behavior of ion selective electrode is usually described by the potentiometric selectivity coefficient (–log K). The potentiometric selectivity coefficients were calculated using the separate solution method, which is recommended by the International Union of Pure and Applied Chemistry (IUPAC) [42]. The logarithms of selectivity coefficient values are given in Table 1. The iodide ion selective electrode based on the functionalized ZnO nanotubes has demonstrated good selectivity towards iodide anion over many of the common organic and inorganic salt anions and the logarithms of selectivity coefficient values were fairly constant. The shelf life of any sensor depends on the conditions where it has to be used and where it has to be kept after use. If both conditions are fully satisfied according to the nature of sensor then it can be used for several days. The proposed iodide ion selective electrode based on functionalized ZnO nanotubes was continuously tested for three weeks and the sensor revealed its good stability, sensitivity, linearity and response time without any abrupt changes in the potential.

**Figure 6.**
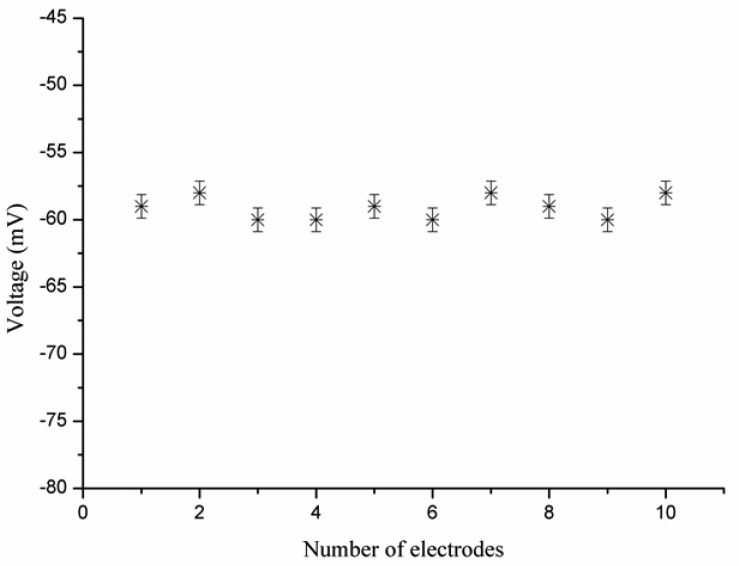
The reproducibility graph of the iodide ion sensor in the 5 × 10^−4^ M solutions of iodide ion.

**Figure 7.**
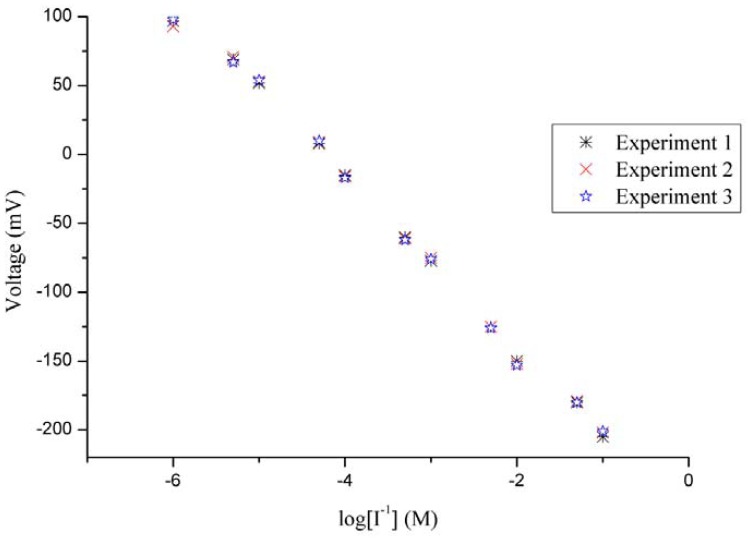
The repeatability calibration curve of the iodide ion sensor.

The evaluation of reproducibility for an ion selective electrode defines its response from one sensor to another sensor under the same working conditions. The reproducibility of 10 iodide ion selective electrodes was checked in 5 × 10^−4^ M solutions of iodide ion. The iodide ion sensor electrodes showed good reproducibility, with a relative standard deviation of less than 8%, as shown in Figure 6. In addition to reproducibility, the repeatability of the iodide ion sensor was also studied. When an independent iodide ion selective electrode was used for three days in the same detectable concentration range of iodide ion, the ion sensor proved itself showing almost same sensitivity, linearity, and response time, as shown in Figure 7.

**Figure 8.**
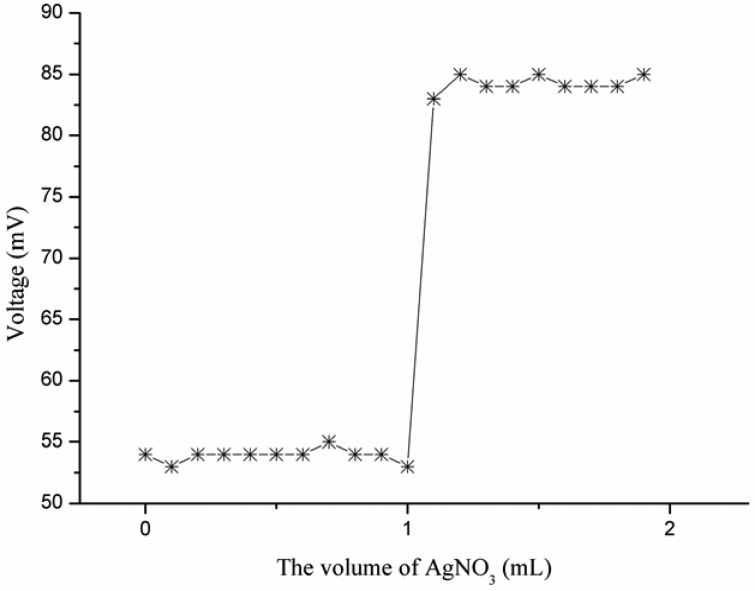
The potentiometric titration curve of iodide ion sensor in the 1 × 10^−4^ M solution of iodide.

The ZnO nanotube-based iodide ion selective electrode was used as an indicator electrode for the determination of iodide using the potentiometric titration method. The iodide ion selective electrode has shown a sharp intersecting point and confirmed its potential applicability as an indicator electrode when titrated in a 1 × 10^−4^ M solution of iodide *vs.* 1 × 10^−2^ M solution of silver nitrate [40], as shown in Figure 8.

## 4. Conclusions

In this work, we have fabricated and grown ZnO nanotubes on a gold coated glass substrate. The developed ZnO nanotube-based iodide ion sensor was functionalised with miconazole nitrate ion exchanger. The iodide ion sensor has shown fast, stable, reproducible and selective behaviour. The functionalised ZnO nanotube-based iodide ion sensor detected a wide range of iodide ion concentrations (1 × 10^−6^ M to 1 × 10^−1^ M) with a sensitivity of −62 ± 1 mV/decade. The detection limit of the iodide ion sensor was found to be 5 × 10^−7^ M. All the obtained results indicate the practical applicability of the proposed iodide ion sensor for the sensing and the detection of iodide ion in real samples.

## Figures and Tables

**Table 1.** The logarithm of selectivity coefficient values.

**Interferents**	**–log K**
Acetate	–3.6
Oxalate	–3.6
Chloride	–3.6
Bromide	–2.1
Nitrate	–3.7
Perchlorate	–3.7
Sulphate	–3.4
Iodate	–3.9
Citrate	–3.2
Ascorbate	–3.6
Cyanide	–3.6
Fluoride	–3.5
Thiocyanide	–3.8
Sulphite	–3.7
Carbonate	–3.6
